# A Survey to Assess the Quality of the Data Obtained by Radio-Frequency Technologies and Microelectromechanical Systems to Measure External Workload and Collective Behavior Variables in Team Sports

**DOI:** 10.3390/s20082271

**Published:** 2020-04-16

**Authors:** Markel Rico-González, Asier Los Arcos, Daniel Rojas-Valverde, Filipe M. Clemente, José Pino-Ortega

**Affiliations:** 1 Departament of Physical Education and Sport, University of the Basque Country, UPV-EHU, Lasarte 71, 01007 Vitoria-Gasteiz, Spain; markeluniv@gmail.com or; 2 Centro de Investigación y Diagnóstico en Salud y Deporte (CIDISAD), Escuela de Ciencias del Movimiento Humano y Calidad de Vida, Universidad Nacional, Heredia 86-3000, Costa Rica; drojasv@una.cr; 3 Escola Superior Desporto e Lazer, Instituto Politécnico de Viana do Castelo, Rua Escola Industrial e Comercial de Nun’Álvares, 4900-347 Viana do Castelo, Portugal; filipe.clemente5@gmail.com or; 4 Instituto de Telecomunicações, Delegação da Covilhã, 1049-001 Lisbon, Portugal; 5 Faculty of Sports Sciences, University of Murcia, 30720 San Javier, Spain

**Keywords:** electronic performance and tracking systems, global position system, local position system, athlete tracking, sensor, MEMS, positioning, time–motion analysis

## Abstract

Electronic performance and tracking systems (EPTS) and microelectromechanical systems (MEMS) allow the measurement of training load (TL) and collective behavior in team sports so that match performance can be optimized. Despite the frequent use of radio-frequency (RF) technology (i.e., global positioning navigation systems (GNSS/global positioning systems (GPS)) and, local position systems (LPS)) and MEMS in sports research, there is no protocol that must be followed, nor are there any set guidelines for evaluating the quality of the data collection process in studies. Thus, this study aims to suggest a survey based on previously used protocols to evaluate the quality of data recorded by RF technology and MEMS in team sports. A quality check sheet was proposed considering 13 general criteria items. Four additional items for GNSS/GPS, eight additional items for LPS, and five items for MEMS were suggested. This information for evaluating the quality of the data collection process should be reported in the methods sections of future studies.

## 1. Introduction

Team sports coaches and scientists use technology with the expectation that it will translate into a competitive advantage [1]. Electronic performance and tracking systems (EPTS) and microelectromechanical systems (MEMS) allow training load (TL) and tactical behavior to be measured at the individual and team level during training and competition [2,3,4,5,6,7,8]. This information is then used to prescribe training sessions, adjust and individualize training programs, and prepare the competition to optimize the performance of the players and the team [9]. 

The main objective of EPTS is to track player (and ball) positioning on the field during training and competition. However, different forms of EPTS have different principles for their use. Global position navigation systems (GNSS)/global positioning systems (GPS) and local positioning systems (LPS) are based on radio-frequency (RF) technology [9,10]. When optic-based methods (VIDs) are used, the objects are tracked by image segmentation using different techniques of image recognition [10]. RF technologies can also be used in combination with microelectromechanical system (MEMS) devices, such as accelerometers, gyroscopes, and heart rate monitors, to measure load or physiological parameters. A chip (i.e., a reception antenna with wireless technology: Bluetooth or Adaptative Network Topology (ANT+)) is incorporated into the device; a chest strap then detects the heart’s electrical signal and sends it to the device. Habitually, EPTS have been used to analyze four kinds of variables in team sports: the physiological analysis (i.e., internal training load (iTL)), time–motion analysis (external workload), neuromuscular analysis, and tactical analysis [6,11,12]. The iTL variables are those that are related to the biological stressors experienced by the player during practice [13]. As we have mentioned, the systems have the possibility of using additional devices to measure these physiological parameters (e.g., HR or oxygen saturation) External workload measures mechanical and locomotor stress during activity and is classified into two groups: kinematic load and neuromuscular load. First, those parameters related to kinematic analysis such as movement patterns, total distances covered or relative distances are obtained through location systems, both LPS and GNSS. Second, neuromuscular analysis is enabled by microelectromechanical sensors (MEMS) such as triaxial accelerometers, gyroscopes, and magnetometers, and includes variables such as accelerations, turns, changes of direction, jumps, player load (the sum of the three-axis acceleration), or impacts [14,15]. Finally, there are the tactical variables that differ from the rest in that, far from quantifying the load, which aim to analyze the positioning and distribution of the players on the field. Even though VIDs have been the most commonly used tool, GNSS/GPS are now widely used, with LPS being the most promising technology [10,11,16]. 

GNSS/GPS calculate the position of players, using a known positioning system (i.e., satellites) as a reference and an object with an unknown position through RF. There are four constellations of satellites (the European Galileo (number of satellites in operation = 22 ); the Russian GLONASS (number satellites in operation = 24); the US-American GPS (nº satellites in operation = 31); and the Chinese Beidou (number satellites in operation = 33)) [17]. A minimum of 24 satellites is needed to obtain useful and valid data during outdoor tracking [18]. In order to calculate the positioning of players, the satellites and receptors have to carry a high-precision synchronized atomic clock. First, a satellite sends a signal at the speed of light to indicate the moment of signal departure, thus allowing the receiver to determine how long the signal has taken to arrive and to multiply the corresponding value by the speed (distance = time × speed). In this way, and by knowing the radius (distance), a sphere is established. The position of the player can be reflected on the earth’s surface at any of the points of the circle/sphere [19]. When a second circle is computed, its position can be at one of the two points where the two spheres intersect on the earth. A third satellite is needed for accurate data to be obtained. Thus, the receiver placed on the player’s back (T2-T4 interscapular), using a specially designed vest, pinpoints the player’s position through this technique, which is called trilateration [14,18,19,20]. Due to the high economic cost of high-precision clocks, the use of a fourth satellite was suggested [19]. The time parameter enters as an unknown variable and is calculated together with the spatial coordinates [19]. For this reason, we find discrepancies regarding the minimum number of satellites needed to calculate the position of the players [21,22].

LPS has been developed as an alternative to GNSS/GPS technology, as unlike GNSS/GPS, it can be used for indoor assessments. Although LPS are based on a unique technology, the principles of its use are quite similar to those of GNSS/GPS [11,16,20]. To compute data with this technology, a set of antennae are used as a reference system instead of satellites. The antennae are placed around the space in which the measurements are taken. The LPS could be classified into infrared, ultrasound and RF (radio-frequency identification, Wi-Fi, Bluetooth, Local Positioning measurement, and ultra-wide-band (UWB)) [16]. 

Most commercially available GPS/GNSS or LPS units contain microsensors that include the use of accelerometers, gyroscopes, and magnetometers, with some commercially available inertial measurement units (IMUs), such as MEMS containing one of or a combination of these sensors [10,23,24,25]. Many researchers have used GPS/GNSS and LPS to quantify the physical demands of sport [14,26], with some also using accelerometers to identify activity profiles [27]. These sensors typically sample at a higher sample rate of up to 500 Hz [20,27]. These sensors can measure the occurrence and magnitude of movement in three dimensions (anterior–posterior, medial–lateral, and vertical). As LPS, IMUs have been applied in elite sporting populations to further understand movement demands [19,26], but unlike GPS/GNSS, the IMUs have the advantage that they can be used indoors as they do not require a satellite connection [20,27]. To date, sports scientists now employ wearable sensors to identify sport-specific movements and activities in an effort to better evaluate the demands of a sport and to assist with physical preparation, injury prevention, and technical analysis of these activities [14,27]. Currently, some studies have assessed the reliability and validity of inertial sensor technology for detecting and assessing sport-specific skills [14,27]. 

Many recent studies have used EPTS and MEMS to assess variables, such as internal and external loads, in different individual and team sports scenarios [28,29,30,31,32]. However, only one study has highlighted the considerations that should be taken into account when utilizing GPS to collect data in a sport setting [20]. In addition, to the best of our knowledge, no study has developed a survey of the use of RF technology and MEMS in practical sports applications. The lack of a survey makes it difficult to compare the results of different studies, complicating systematic reviews and meta-analyses. Thus, this study aimed to develop the first survey for collecting, processing, and reporting GNSS/GPS, LPS, and MEMS data. We hypothesized that there would be a lack of information on the method in the articles in which RF technology was used. 

Related publications: This work is motivated by the fact that GPS/GNSS, LPS, and MEMS are widely used in sport for positioning and tracking, and the research related to them is still an active and open research area [15,16,20,26,27,33]. In research, detailed reporting standards are considered necessary in the field of measurement to ensure that outputs conform to standards for reporting trials (CONSORT) or observational studies (STROBE) [20]. This paper is an extended version of papers published about principles of use collected from the literature [11,14,15,16,18,19,20,27,33,34,35,36,37,38,39] that could affect the quality of the data obtained during sport locomotion and tracking assessments. 

## 2. Evaluation Methodology: Criteria

The criteria were divided into two groups: (1) general criteria, which are valid for GNSS/GPS, LPS, and MEMS, and (2) a group of specific criteria for each of them. Firstly, a check sheet of 13 general criteria items was suggested (Table 1). Together with the general criteria, several specific criteria were suggested for the Global Navigation Satellite System/Global Positioning System (Table 2), for Local Positioning Systems (Table 3), and for microelectromechanical systems (MEMS) (Table 4). 

### 2.1. General Criteria

#### 2.1.1. Reliability and Validity

In order to utilize the output of a system for player monitoring, the data should be both valid and reliable [35]. A high level of consistency among the measurements recorded by a system indicates that it is able to reliably detect meaningful changes in an athlete’s performance [40]. All technologies are prone to some percentage of error. Due to inevitable errors, there is a need to explore the accuracy of these technologies under various sport environments. Due to inevitable errors, there is a need to explore the accuracy of these technologies under various sport environments. These errors are the responsibility of the manufacturer. Hence, the errors commented on so far are the responsibility of the manufacturer. Hence, the sports scientist should ensure that the technology used is reliable and valid. In fact, the technology is continually improving through developments related to microprocessors, data processing, and software. Additionally, new models/brands sometimes differ in terms of sampling rates, chipsets, filtering methods, and data processing algorithms. For these reasons, sports scientists are continuously investigating whether the reliability and accuracy of each device are acceptable [20]. 

Tests for accuracy will help to guarantee the optimal use of these technologies by coaches, athletes, and other staff. Planimetry, calibration [36], external factors during the signal travel time, or multipath are examples of these problems [16]. Planimetry positioning refers to the combination of *x*- and *y*-coordinates on a plane and is understood as the distance between the recorded position and the real position. The aging and manufacture of the sensors lead to calibration error (i.e., misalignment and a lack of orthogonality of the axes). Scale calibration can affect the data of the gyroscope and the accelerometer. It behaves like a bias error when integrating the signal, and the error accumulates because of the temperature during the time when the device is functioning [41]. On the other hand, although the measurement method assumes that the signal’s velocity is constant, when the signal goes through the biosphere and troposphere, it suffers delays and the distance data assume errors. Finally, the multipath condition has become a problem and, as yet, has no solution. However, due to the large data rate, bandwidth, and extremely short pulses, waveforms allow UWB to reduce the effect of multipath interference [16]. 

The sport or variable in which the data will be recorded should be considered because validity and accuracy processes are expected to be different for different sports. In fact, when scientists try to assess variables considering multiple players (i.e., collective positioning), the inter-unit reliability should be assessed [24]. However, if a scientist wants to record individual variables, inter-unit reliability is not necessary [20,42]. Linke et al. [10] showed that validity and reliability studies can be divided into three categories: (1) studies that analyze position accuracy, (2) studies that analyze speed and acceleration data, and (3) studies that propose continuous situations such as real conditions. The accumulative error associated with the first and second categories can lead to errors in the last one [10]. So, even though there is no standard system that provides perfect accuracy, authors should cite articles in which their brand/model has been assessed in continuous situations. Finally, it is important to note that for GPS/GNSS and LPS alike, the magnitude of the error increases at the peak acceleration and deceleration of the tracking objects [10,43]. However, MEMSs have allowed the quantification of high-speed running with great accuracy without RF signals [40].

#### 2.1.2. Sampling Frequency

An important parameter used to assess variables is the amount of data recorded per second, namely Hertz (Hz) [35]. In fact, the sampling frequency is related to the accuracy of the technology both in terms of acceleration or velocity [43] and positioning variables [26]. As such, the technology is not useful if the Hz is not considered before each field study. Firstly, the frequency depends on the capacity of the positioning systems used in the intervention. Secondly, it depends on the decision of the researcher or sports technician who can configure tools to extract more or less data from the session [26]. It is crucial that the Nyquist theorem is respected. That is, the sampling frequency must be at least twice as high as the highest frequency given by the signal itself. In addition, sports technicians should consider that if the sampling frequency is too low, there will be errors in the recording [43] and that if it is too high, noise could contaminate the desired signal [16,44,45]. On the other hand, software-derived data should be considered carefully [20]. Manufacturers’ software often includes algorithms that can be used to identify poor-quality data. Researchers can modify the frequency of the data, and the software will automatically interpolate, smooth, or extract software-derived data [20]. A greater Hz value is correlated with higher sampling frequencies, which will not necessarily yield better results [26]. So, although data per unit of time could be dependent on each variable, further studies should analyze the influence of the raw data and software-derived data on the measurement of kinematic, physiological, or tactical variables. 

To normalize the data and avoid the type of noise mentioned above, the accelerometer chip manufacturer usually applies a first filtration process. The filtration details are not commonly specified by the device fabricant, and the user cannot change the chip configuration. In order to better understand this process, the manufacturers could give more information about the filtration stages in the chip, software, and user options. This information could give the user valuable knowledge about how the data is collected and processed before the “raw data” is made available for the user. This would allow the sports scientist to make inter-device comparisons and, in turn, to understand the main differences among them.

#### 2.1.3. Computation Methods for Velocity and Acceleration

RF tracking systems have the potential to measure several variables. These variables must be positioned at three levels to accommodate the calculation methods. At the first level is the positioning, which is calculated as a direct variable. At the second level is the velocity, which serves as an indirect variable. At the third level, the velocity can be used to calculate acceleration and deceleration. GNSS/GPS or LPS can calculate the distance and the velocity from positional differentiation or frequency difference of arrival (differential Doppler) [16,20]. First, the algorithm computes the velocity or acceleration data using the distance information from each satellite (GNSS/GPS) or local antennae (LPS) and then using the triangulation device position. Finally, the distance or acceleration is derived from the change in positioning with each signal. In the second case, velocity and acceleration data are extracted as a means of measuring the changes in the frequency of the periodic signal emitted by the reference system. Researchers should include this information in their methods because of the higher accuracy of the calculated distance when the Doppler shift is used as opposed to positional differentiation [46].

#### 2.1.4. Exclusion/Inclusion Criteria

Due to factors beyond the practitioner’s control, there can be moments in which data should be eliminated from the analysis. Raw traces of velocity and acceleration should be checked to detect spikes or outliers in the data generated from the technology itself. These irregularities could be justified by a sudden loss in the satellite signal connection, leading the detection of data to be delayed. In fact, the number of satellites and high dilution of precision (HDOP) values could be used for a criterion to delete data [20]. Therefore, researchers are encouraged to detail the specific procedure and filtration processes that were considered when extracting the data. Performing this task will clarify which exclusion criteria were used to avoid signal bias.

#### 2.1.5. Real-Time vs. Post-Game Data

Currently, international sport regulations allow technology in the technical area to track in real time. In some sports, such as Australian football, the use of EPTS is allowed during matches [37]. However, there is a debate about the difference in accuracy between data downloaded during a match or training session and data downloaded after a match or training session. Among the advantages of using GNSS/GPS or LPS for real-time data are: (1) the possibility of recording multiple players at the same time, (2) the time effectiveness of the analysis, and (3) the possibility of giving feedback in real time and making fast and opportune decisions [47]. However, when real-time data are to be used, some factors should be considered, such as: (1) where and how the calculation is going to be made (i.e., within devices or on an external PC); (2) system delay, coverage technology, or technology used; (3) the use of a necessary infrastructure that does not affect the signal; (4) the number of devices that can be displayed at the same time; (5) the number of variables and whether choosing them is possible; and (6) the possibility of setting alarms to provide feedback quickly. On the other hand, when data are extracted after the session, other considerations must be taken into account, including: (1) whether the system allows raw data extraction; (2) the download time; and (3) whether the software allows the user to manage and analyze data freely. Aughey and Falloon [37] investigated whether data can change depending on whether they are downloaded in real time or post-session. Although the correlations between real-time data and post-game data were strong for all parameters, the difference in the mean and total error was large with a wide range of scores. The results showed that caution is needed when the data have been downloaded in real time, especially regarding high-intensity effort. Usually, the data is downloaded after training or the match when the aim of the data collection is to publish an article. This is consistent with other research in which the data monitored in real time were significantly inaccurate relative to the post-session data for the external load parameters of maximum velocity, overall distance covered, high-speed distance, high-intensity activity, and sprint distance [48]. Thus, research articles should mention the moment at which the researchers download the data. 

#### 2.1.6. Technology Lock

Whether GNSS/GPS or LPS should be used depends on the reference system connected to the devices. In order to avoid GPS lock, researchers must ensure that the receptors have satellite or antennae connections before the training session or match starts. This can be achieved by placing the devices in clear outdoor space (for GNSS/GPS devices) or in the middle of the antennae system, which is usually indicated on the manufacturer’s device by flashing light signals [20]. Duffield, Reid, Baker, and Spratford [49] reported that GPS/GNSS should be activated 15 min prior to data collection to allow for the acquisition of satellite signals. 

#### 2.1.7. Data Synchronization for Collective Tactical Analyses

Even though previous studies have recorded raw data and then reduced them with different techniques (i.e., smooth, Butterworth filter, cut-off frequencies) to extract software-derived data, the accuracy of the positional information that is used to determine the distance between multiple units is different from the precision of individual variables [20]. To record collective variables when GNSS/GPS technology is used to record data, the satellite’s atomic clock sends a signal, and the receptor records the data and the time at which the signal was sent. Therefore, as there is no common clock that is shared by the devices, the data can be recorded at different times. Even though LPS involve a common clock, this problem can still occur [50]. For example, the LPS positioning sensor of the device used 18 Hz, which correlates with the recording of one data point every 55.55 milliseconds, with each device consistently recording that amount of data. However, each device obtains the data within that time window, which means that the data may not coincide in time, with a maximum difference of 55.54 milliseconds. Data synchronization methods are wide, and researchers should consider at least one of them in their studies [51].

### 2.2. Specific Criteria for Global Navigation Satellite System/Global Positioning System

#### 2.2.1. Number of Satellite Connections and HDOP. 

The signals received by devices from satellites influence the accuracy of the data recorded, and the signal quality may change depending on the place where the field study is done [20]. An assessment of whether the signal is acceptable can be based on two parameters: (1) the number of satellites connected to the device and (2) the orientation of the satellites in the atmosphere [19]. Although these parameters vary during the session and it is difficult to report exact conditions, the mean conditions should be reported [18,46]. Although only three satellites are required for trigonometry, a fourth satellite is necessary to eliminate the need for expensive clocks [19]. Thus, a minimum of four satellites is needed for the level of connection of a system to be deemed adequate. However, the use of additional satellites is recommended to ensure the coverage of large areas. Malone et al. [20] anecdotally report that if the GPS/GNSS receiver is connected to less than six satellites, the connection tends to be weak, and the data tend to be of poor quality. However, Linke et al. [10] reported that the numbers of satellites connected in validation studies were 8 ± 1, 9.5 ± 2, and 12.3 ± 0.3. Although further studies are needed to confirm this, manufacturers should consider that the number of constellations used whose satellites are connected with devices could influence the area covered and the number of satellites connected with devices [18]. 

On the other hand, the horizontal dilution of precision (HDOP) refers to the geometrical organization of the satellites in the atmosphere and could influence the accuracy of the data obtained. The HDOP value is higher the closer the satellites are to each other, and high HDOP value is associated with poor-quality data. So, the greater the dispersion of the satellites, the better the quality of the reported data, with an HDOP value of less than one considered optimal [20]. As has been mentioned, GNSS can yield a lower HDOP value than GPS, which uses only one constellation (i.e., American).

#### 2.2.2. Environmental and Infrastructure Conditions. 

GNSS/GPS were initially proposed for military use in outdoor environments. However, indoor positioning systems have recently been developed to replace GNSS/GPS in indoor environments. GNSS/GPS signal quality can be influenced by infrastructure (e.g., houses, walls) or the weather, which means that the data used to report study results cannot be recorded on adequate fields or days. In fact, GNSS/GPS are suitable and efficient for outdoor environments or places without tall buildings, as opposed to indoor environments or stadiums because the satellite radio signal cannot penetrate solid walls, curved roofs, or obstacles [16] and can provide unreliable data because fewer satellites are available to triangulate signals from devices [14]. Therefore, authors must explain these conditions in their methods.

### 2.3. Specific Criteria for Local Positioning Systems

#### 2.3.1. Environmental and Infrastructure Conditions

As has been mentioned, when LPS are used, a reference system is installed around the court. Low temperatures, humidity gradients, and slow air circulation can allow for easier positioning within this small area [16]. Although many technologies are not useful because of their sampling frequency capacity or interference with several multipaths (i.e., ultrasound) [11], different kinds of technology have been used to measure different variables [4,42]. One of these types of technology is UWB, which is still subject to interference caused by metallic materials.

#### 2.3.2. Installation

Unlike GNSS/GPS, when LPS is used, the antennae installation shape and height must be considered because these factors can influence the final data. The scientist must consider that each antenna has an error margin around it, like a circumference. The antennae are installed around the court. The antennae in the corners are the closest to the court lines, while the antennae located in the middle of the court and (when eight antennae are installed) behind the goals are farther away from the court lines [52]. This means that the error margins are not as prominent inside the court where the players tend to run the most. So, although it is difficult in some places, the optimal shape of the antennae installation is a circumference [53], and if this circumference is deformed along the lateral or longitudinal axes, the accuracy of the *x* or *y* data, respectively, will decrease. Moreover, although it is common to install the antennae around the court, if teams want to install fixed antennae, they must consider that the higher the antennae are, the more error-prone they are. Thus, it is suggested that future studies provide details about the shape and height of the installed antennae.

#### 2.3.3. Measurement Method

UWB is a very promising technology. Different positioning measurement methods have been applied to report data from RF signals between the antennae and devices. The high number of positioning algorithms can be classified into five main categories based on estimated measurements [16]: (1) time of arrival (TOA); (2) angle of arrival (AOA); (3) received signal strength (RSS); (4) time difference of arrival (TDOA), and (5) a hybrid algorithm. An understanding of the accuracy, environment, estimation technique, space, and purpose of use of these algorithms is critical because of their differences and the appropriateness of their use in different situations. 

AOA is less practical than the other four types of algorithms because of the difficulty and cost of maintaining the required large dimensions of antenna arrays and sensors. Moreover, this algorithm requires a high level of cooperation among the sensors and is subject to error accumulation [54]. Although this method has acceptable precision, AOA and RSS are more suitable than other algorithms for systems that use narrowband signals with a high UWB bandwidth [16]. Because of its suitability for the narrowband method, RSS is less attractive than other measurement methods that allow great accuracy. However, TOA performs better in wideband systems, like UWB [16]. In terms of accuracy, small errors in AOA will negatively impact precision when the target object is far away from the base station. However, TOA and TDOA are more accurate relative to other algorithms because of the high time resolution of the UWB signals. Clock synchronization and clock jitter are important factors that affect the accuracy of TOA because, as mentioned earlier in this study, clock synchronization is needed among the receivers to estimate TOA’s precision. However, TDOA is a more effective option if there is no synchronization between the receivers and antennae when the reference nodes are synchronized among themselves [55]. Hybrid algorithms have been found to be the most effective solutions for UWB positioning systems because they combine the advantages of all the algorithms [16]. 

### 2.4. Specific Criteria for Microelectromechanical Systems (MEMS)

#### 2.4.1. Minimum Effort Duration

From the practical point of view of a sports scientist, a data processing feature should be considered to be able to customize by software when the scientist wishes to analyze high-intensity movement efforts, such as sprints or accelerations [44]. In fact, with the technology that is currently available, it is likely that unrealistic calculations of such efforts will be made. For example, GPS random error or spikes in speed could occur. However, the scientist can avoid such errors by inserting minimum effort duration into the software [20]. Two parameters are necessary to compute the minimum effort duration: time and velocity. For example, if the GNSS/GPS or LPS is computed at 10 Hz for the raw data and the detection of a sprint effort is defined as >7 m/s for a minimum time of 0.5 s, the device requires the speed to be maintained at >7 m/s for a minimum of five consecutive samples (i.e., 10 Hz = 10 samples per second). In this respect, five samples will be produced in 0.5 s. The identification of the final moment is also important, as speed may oscillate around a set threshold. Therefore, a minimum time in which speed is required to fall below a threshold should also be determined. For example, an athlete’s speed may oscillate around the sprint threshold of 7 m/s. If a short minimum time is used to detect the end of an effort (e.g., 0.1 s), then if the athlete’s speed fell below the threshold for one sample, he/she would be reported to have performed two or more sprint efforts when only one effort was made [20]. However, further studies should be carried out to determine the optimal duration to avoid the abovementioned parameters. However, too short a duration can result in an inaccurately high number of efforts being reported. Moreover, the minimum duration used to identify the start and end of an effort can have an especially large effect on the identification of short-duration efforts, such as accelerations and decelerations. A conservative approach for users would be to set a duration that is longer than a given threshold, such as the criteria for accelerations and decelerations. Practitioners should be aware that these user-defined criteria may have a marked effect on their results and should be consistent with their choice of minimum time [20]. In addition, differences among studies in terms of the criteria used to define efforts (or the absence of defined criteria) make it difficult to compare findings. For these reasons, new studies should include in their methods the parameters discussed in the following subsections.

#### 2.4.2. Clothes

MEMSs have great potential for detecting sport-specific movements and can quantify sporting demands that other devices might not detect; however, they are highly sensitive [56,57]. For this reason, participants should carry the receptors in appropriate tight-fitting garments to avoid unwanted movements and, subsequently, poor data. Moreover, these microsensors must be secured in the same position for all sessions. This suggestion is based on the movements the players make when using match jerseys with custom-made pouches sewn into the back, which may differ from training jerseys, and researchers should explain in future articles that athletes should wear the same garment in routine training that they wear during competition [20].

## 3. Experimental Results

This study aimed to develop the first survey for collecting, processing, and reporting GNSS/GPS and LPS data. The survey considered the following criteria: reliability, validity, sampling frequency, computation methods for velocity and acceleration, data exclusion and inclusion criteria, high-intensity bias due to random error, the time at which the data were extracted, technology lock, and data synchronization. It also took into account the athlete’s clothes, the number of reference points and satellites, environmental and infrastructure conditions, antennae installation and position, and measurement methods. In light of the abovementioned considerations, a survey was proposed. This criterion was embodied in a check sheet of 13 general criteria items (Table 1), along with four additional items for GNSS/GPS (Table 2), eight additional items for LPS (Table 3), and five additional items for MEMS (Table 4). Based on these criteria, several recently published articles were assessed using the new survey (Table 5).

Results showed that no study surpassed 45% in the assessment of the quality of the data. Thus, as we hypothesized, there is a lack of information about the collecting, processing, and reporting of GNSS/GPS, LPS and MEMS data (Table 5). Therefore, this information should be reported in the methods sections of future studies due to its impact on the quality of the results obtained during data collection, data processing, data analysis, and data collection, processing, analysis, and reporting.

## 4. Concluding Remarks

Considering that there are several principles of use as well as general and specific criteria that could affect the quality of the data obtained during sport locomotion and tracking assessments, a new survey has been suggested to serve as a guideline for researchers and sports scientists. Some considerations have been addressed to avoid data error, such as those pertaining to reliability, validity, sampling frequency, computation methods for velocity and acceleration, data exclusion and inclusion criteria, high-intensity bias due to random error, the time at which the data were extracted, technology lock, and data synchronization. Other factors such as the athlete’s clothes, the number of reference points and satellites, environmental and infrastructure conditions, antennae installation and position, and measurement methods have also been mentioned. This information should be reported in the methods sections of future studies due to its impact on the quality of the results obtained during data collection, data processing, data analysis, and data collection, processing, analysis, and reporting.

## Figures and Tables

**Table 1:** Check sheet for assessing external workload and collective variables using radio-frequency technologies (i.e., GPS/GNSS and LPS) in sports.

Quality Index Item Number	Criteria			
GC	*General criteria*			
GC1	Was the process to avoid technology lock explained?	Yes = 1	No = 0	
GC2	Was the data download moment mentioned?	Yes = 1	No = 0	
	*Reliability and validity*			
GC3	Was the brand/model mentioned?	Yes = 1	No = 0	
GC4	Were the variability and reliability of the model cited?	Yes = 1	No = 0	
GC5	Was the model assessed for variability or reliability according to the variables used? (Multi-player vs single player)	Yes = 1	No = 0	
GC6	How was the validation test performed?	Continuous situations = 2	No continuous situations = 1	Not cited = 0
GC7	Were data exclusion criteria mentioned?	Yes = 1	No = 0	
GC8	Was a sensor fusion algorithm explained (only for velocity and acceleration)?	Yes = 1	No = 0	
	Sampling frequency			
GC9	Was the raw data justified?	Yes = 2	No = 1	No appearance = 0
GC10	Was the software-derived data justified?	Yes = 2	No = 1	No appearance = 0
GC11	Was a data reduction method mentioned?	Yes = 1	No = 0	
GC12	Were different Hz values used for each variable reported?	Yes = 1	No = 0	
GC13	Was the time synchronisation method explained (only for collective measures (i.e. tactical variables)?	Yes = 1	No = 0	

**Table 2:** Check sheet for assessing external workload or collective variables using GPS/GNSS in sports.

Quality Index Item Number	Criteria		
GPS1	Was the satellite number mentioned?	Yes = 1	No = 0
GPS2	Were the HDOP values mentioned?	Yes = 1	No = 0
GPS3	Were weather conditions reported?	Yes = 1	No = 0
GPS4	Was the infrastructure around the field described?	Yes = 1	No = 0
• Total score for distance covered / out of 18			
• Total score for velocity / out of 19			
• Total score for collective tactical behavior / out of 19			
• Percentage score/ %			

**Table 3:** Check sheet for assessing external workload or collective variables using LPS in sports.

Quality Index Item Number	Criteria		
LPS1	Was the technology mentioned (e.g. UWB, ultrasounds)?	Yes = 1	No = 0
LPS2	Was the temperature reported?	Yes = 1	No = 0
LPS3	Were humidity gradients reported?	Yes = 1	No = 0
LPS4	Was it mentioned whether there was slow air during the sessions?	Yes = 1	No = 0
LPS5	Was it mentioned whether there were any metallic materials around the antennas?	Yes = 1	No = 0
LPS6	Was the installation shape explained?	Yes = 1	No = 0
LPS7	Was the installation height reported?	Yes = 1	No = 0
LPS8	Was the measurement method reported?	Yes = 1	No = 0
• Total score for distance covered / out of 22			
• Total score for velocity / out of 23			
• Total score for collective tactical behavior / out of 23			
• Percentage score/ %			

**Table 4:** Check sheet for assessing external workload or collective variables using MEMS in sports.

Quality IndexItem Number	Criteria		
MEMS1	Were algorithms for velocity and acceleration mentioned?	Yes = 1	No = 0
MEMS2	Was an algorithm for velocity or acceleration mentioned?	Yes = 1	No = 0
MEMS3	Were the minimum effort duration and minimum speed for avoiding unrealistic data mentioned?	Yes = 1	No = 0
MEMS4	Was it mentioned whether the participants wore tight-fitting garments?	Yes = 1	No = 0
MEMS5	Was it mentioned whether the participants wore the same garment?	Yes = 1	No = 0
• Total score/ out of 20			
• Percentage score/ %			

**Table 5:** Practical application of the survey in the different types of analyses with different technologies.

Global Navigation Satellite System																										
Ref.	Analyze Type	Variables	GC1	GC2	GC3	GC4	GC5	GC6	GC7	GC8	GC9	GC10	GC11	GC12	GC13	GNSS1	GNSS2	GNSS3	GNSS4					From*	TS	%
[38]	Cinematic	-Dist. covered -Speed	1	0	1	1	1	1	0	0	1	0	0	0	-	1	0	1	0					20	8	40
[51]	Cinematic	-Dist. covered -Dist. at different intensities	1	0	1	1	1	2	0	-	1	0	0	0	-	1	0	0	0					18	8	44
[58]	Cinematic	-Dist. covered -Dist. at different intensities	0	0	1	0	0	0	0	-	1	1	1	0	-	0	0	0	0					18	4	22
[51]	Tactic	-Dyads	1	0	1	1	0	0	0	-	1	0	0	0	1	1	0	0	0					19	6	32
[52]	Tactic	-Dyads -Area	0	0	1	0	0	0	0	-	1	1	1	0	0	0	0	0	0					19	4	21
*Local Positioning Systems*																										
Ref.	Analyze type	Variables	GC1	GC2	GC3	GC4	GC5	GC6	GC7	GC8	GC9	GC10	GC11	GC12	GC13	LPS1	LPS2	LPS3	LPS4	LPS5	LPS6	LPS7	LPS8	From	TS	%
[59]	Cinematic	-Dist. covered -Dist. at different intensities -Nº of sprint	0	0	1	1	1	2	0	-	1	0	0	0	-	0	0	0	0	0	0	0	0	22	6	27
[59]	Tactic	-GC -Dyads -Area	0	0	1	1	1	0	0	-	1	0	0	0	0	0	0	0	0	0	0	0	0	23	4	17
*Micro-Electromechanical Systems*																										
Ref.	Analyze type	Variables	GC1	GC2	GC3	GC4	GC5	GC6	GC7	GC8	GC9	GC10	GC11	GC12	GC13	MEMS1	MEMS2	MEMS3	MEMS4	MEMS5				From	TS	%
[60]	Neuromuscular	-Accelerometry based variables	1	0	1	1	1	2	0	0	1	1	0	0	-	0	0	0	1	0				20	9	45
[61]	Neuromuscular	-Impacts -Steps -Jumps	0	1	1	1	0	0	0	0	0	0	0	0	-	0	0	1	1	0				20	5	25
*The total number of points for calculation is based on the technology used and type of variable (velocity, distance covered, tactical or neuromuscular); TS: total score; % percentage; “- “: no applicable																										
